# Supplementary material for: Risk factors for early suspected ventilator-associated pneumonia in severe thoracic blunt trauma patient: A French national cohort study
Source: PLoS One. 2025 May 27;20(5):e0324120. doi: 10.1371/journal.pone.0324120 (PMC12112400; doi:10.1371/journal.pone.0324120)
Supplement: S1 Table — (DOC) [file pone.0324120.s002.docx]

Supplemental table : Descriptive statistics and univariate analysis comparing the suspected VAP and non-suspected VAP group. Quantitative variables are expressed in medians and interquartile ranges (25-75) or in means ± standard deviations depending on their distribution.

| Variable | Sample size | Total population |
| --- | --- | --- |
| Age (years) | 712 | 39±17 |
| Weight (kgs) | 647 | 77±16 |
| Height (m) | 617 | 1.75±0.05 |
| BMI | 615 | 25±4 |
| Male sex | 712 | 574 (80%) |
| ASA score | 701 | 1.6±1.2 |
| ASA score | 685 |  |
| *1* |  | 457 (65%) |
| *2* |  | 184 (26%) |
| *3* |  | 26 (4%) |
| *4* |  | 5 (<1%) |
| *5* |  | 6 (<1%) |
| *6* |  | 7 (<1%) |
| ASA score >1 | 701 | 244 (35%) |
| IGS Score | 712 | 45±16 |
| SOFA respiratory system | 692 | 1.8±1.3 |
| SOFA coagulation | 688 | 0.60±0.84 |
| SOFA cardiovascular system | 690 | 3.0±1.5 |
| SOFA Central nervous system | 691 | 2.0±1.5 |
| SOFA Liver | 670 | 0.23±0.56 |
| SOFA renal function | 688 | 0.30±0.59 |
| Total SOFA Score | 712 | 8.3±3.5 |
| SOFA respiratory system | 712 |  |
| *0* |  | 166 (24%) |
| *1* |  | 130 (19%) |
| *2* |  | 152 (22%) |
| *3* |  | 166 (24%) |
| *4* |  | 78 (11%) |
| SOFA coagulation | 712 |  |
| *0* |  | 415 (60%) |
| *1* |  | 149 (22%) |
| *2* |  | 109 (16%) |
| *3* |  | 13 (2%) |
| *4* |  | 2 (<1%) |
| SOFA Cardiovascular system | 712 |  |
| *0* |  | 118 (17%) |
| *1* |  | 31 (4%) |
| *2* |  | 2 (<1%) |
| *3* |  | 111 (16%) |
| *4* |  | 428 (62%) |
| SOFA Central nervous system | 712 |  |
| *0* |  | 97 (14%) |
| *1* |  | 280 (41%) |
| *2* |  | 76 (11%) |
| *4* |  | 238 (34%) |
| SOFA liver | 712 |  |
| *0* |  | 561 (84%) |
| *1* |  | 69 (10%) |
| *2* |  | 39 (6%) |
| *4* |  | 1 (<1%) |
| SOFA renal function | 712 |  |
| *0* |  | 525 (76%) |
| *1* |  | 127 (18%) |
| *2* |  | 33 (5%) |
| *3* |  | 1 (<1%) |
| *4* |  | 2 (<1%) |
| Total SOFA SCORE | 712 |  |
| *0* |  | 26 (2%) |
| *1* |  | 9 (1%) |
| *2* |  | 15 (2%) |
| *3* |  | 29 (4%) |
| *4* |  | 39 (5%) |
| *5* |  | 45 (6%) |
| *6* |  | 68 (10%) |
| *7* |  | 62 (9%) |
| *8* |  | 81 (11%) |
| *9* |  | 64 (9%) |
| *10* |  | 84 (12%) |
| *11* |  | 69 (10%) |
| *12* |  | 56 (8%) |
| *13* |  | 40 (6%) |
| *14* |  | 19 (3%) |
| *15* |  | 8 (1%) |
| *16* |  | 6 (<1%) |
| *17* |  | 3 (<1%) |
| *20* |  | 1(<1%) |
| AIS head | 712 | 2.6 ± 1.8 |
| AIS face | 712 | 0.76±1.05 |
| AIS Chest | 712 | 3.5±0.6 |
| AIS abdomen | 712 | 1.2±1.5 |
| AIS extremities | 712 | 1.9±1.4 |
| AIS external | 712 | 0.2±0.4 |
| AIS Head | 712 |  |
| *0* |  | 178 (25%) |
| *1* |  | 21 (3%) |
| *2* |  | 92 (13%) |
| *3* |  | 183 (26%) |
| *4* |  | 139 (19%) |
| *5* |  | 104 (15%) |
| AIS Face | 712 |  |
| *0* |  | 431 (60%) |
| *1* |  | 92 (13%) |
| *2* |  | 133 (19%) |
| *3* |  | 57 (8%) |
| *4* |  | 4 (<1%) |
| AIS Chest | 712 |  |
| *3* |  | 452 (63%) |
| *4* |  | 206 (29%) |
| *5* |  | 59 (8%) |
| AIS Abdomen | 712 |  |
| *0* |  | 379 (53%) |
| *1* |  | 7 (1%) |
| *2* |  | 184 (26%) |
| *3* |  | 90 (13%) |
| *4* |  | 38 (5%) |
| *5* |  | 19 (3%) |
| AIS Abdomen >2 | 712 | 147 (21%) |
| AIS extremities | 712 |  |
| *0* |  | 203 (28%) |
| *1* |  | 28 (4%) |
| *2* |  | 220 (31%) |
| *3* |  | 202 (28%) |
| *4* |  | 40 (6%) |
| *5* |  | 24 (3%) |
| AIS external | 712 |  |
| *0* |  | 572 (80%) |
| *1* |  | 144 (20%) |
| *2* |  | 1 (<1%) |
| ISS score | 712 | 31±11 |
| ISS score >15 | 712 | 678 (95%) |
| Revised trauma score | 636 | 5.9±1.5 |
| Trauma injury severity score | 636 | 0.29±0.28 |
| Mechanism of injury | 712 |  |
| *Road accident* |  | 520 (73%) |
| *Fall* |  | 174 (24%) |
| *Blunt force trauma* |  | 12 (2%) |
| *Other* |  | 11 (2%) |
| Fall height | 167 |  |
| *<6m* |  | 47 (28%) |
| *>6m* |  | 120 (72%) |
| Transport vector | 712 |  |
| Helicopter |  | 183 (26%) |
| Truck |  | 532 (74%) |
| Blast | 587 | 1 (<1%) |
| Limb ischemia | 710 | 23 (3%) |
| Amputation | 710 | 13 (2%) |
| Broken pelvis | 709 | 58 (8%) |
| Burn injury | 712 | 1 (<1%) |
| Anticoagulent treatment | 704 | 6 (<1%) |
| Antiplatelet drug | 704 | 19 (3%) |
| Initial loss of consciousness | 193 | 83 (43%) |
| Initial GCS | 709 | 8.9±4.4 |
| Initial GCS | 709 |  |
| *3* |  | 124 (17%) |
| *4* |  | 32 (5%) |
| *5* |  | 41 (6%) |
| *6* |  | 74 (10%) |
| *7* |  | 54 (8%) |
| *8* |  | 45 (6%) |
| *9* |  | 41 (6%) |
| *10* |  | 25 (4%) |
| *11* |  | 20 (3%) |
| *12* |  | 31 (4%) |
| *13* |  | 44 (6%) |
| *14* |  | 66 (9%) |
| *15* |  | 112 (16%) |
| Initial motor GCS | 665 | 4.1±2.0 |
| Initial Motor GCS | 665 |  |
| *1* |  | 143 (22%) |
| *2* |  | 29 (4%) |
| *3* |  | 53 (8%) |
| *4* |  | 91 (14%) |
| *5* |  | 88 (13%) |
| *6* |  | 261 (39%) |
| Initial GCS <9 | 709 | 370 (52%) |
| Initial GCS <12 | 709 | 456 (64%) |
| Inital SBP | 675 | 123±30 |
| Inital DBP | 674 | 74±20 |
| Initial HR | 677 | 101±28 |
| Inital SpO_2_ | 180 | 95±6 |
| Minimal SBP during transport | 641 | 99±25 |
| Minimal DBP during transport | 639 | 59±18 |
| Maximal HR during transport | 642 | 111±27 |
| Minimal SpO2 during transport | 648 | 92±10 |
| Initial Hemocue | 605 | 13±2 |
| Hemocue at hospital arrival | 666 | 12±2 |
| Pre hospital Cristalloïds (mL) | 635 | 1035±576 |
| Pre hospital Colloids (mL) | 642 | 29±142 |
| Prehospital catecholamine infusion | 706 | 259 (37%) |
| Prehospital maximal Catecholamines dosage (mg/h) | 64 | 2.2±1.8 |
| Prehospital PRBC | 712 | 0.06±0.37 |
| Prehospital FFP | 712 | 0.00±0.07 |
| HR upon arrival at hospital | 710 | 99±25 |
| SBP upon arrival at hospital | 708 | 115±40 |
| DBP upon arrival at hospital | 708 | 71±22 |
| Catecholamines infusion upon arrival at the hospital | 712 | 70 (10%) |
| Catcholamines dosage upon arrival at the hospital | 68 | 2.5±2.4 |
| Respiratory rate upon arrival at the hospital | 163 | 19±6 |
| SpO_2_ upon arrival at the hospital | 700 | 98±6 |
| EtCO_2_ upon arrival at the hospital | 91 | 36±10 |
| Temperature upon arrival at the hospital | 661 | 35.9±1.4 |
| pH upon arrival at the hospital | 683 | 7.27±0.11 |
| PaO2 upon arrival at the hospital | 682 | 209±128 |
| PaCO_2_ upon arrival at the hospital | 684 | 46±11 |
| Lactates upon arrival at the hospital | 693 | 2.8±2.6 |
| FiO_2_ upon arrival at the hospital | 440 | 0.62±0.21 |
| Hemoglobin upon arrival at the hospital | 712 | 12±2 |
| Platelet upon arrival at the hospital | 710 | 227595±78243 |
| Prothrombin Ratio upon arrival at the hospital | 710 | 72±18 |
| Quick Time Ratio upon arrival at the hospital | 141 | 1.3±0.6 |
| Fibrinogen upon arrival at the hospital | 702 | 2.1±0.9 |
| Creatinine upon arrival at the hospital | 712 | 89±43 |
| HCO_3_- upon arrival at the hospital | 702 | 20±4 |
| Troponins upon arrival at the hospital | 90 | 43±106 |
| Ultrasensitive troponins upon arrival at the hospital | 602 | 187±821 |
| Blood alcohol level upon arrival at the hospital (g/L) | 605 | 0.55±0.88 |
| Prehospital transfusion | 712 | 30 (4%) |
| PC before scanner | 712 | 0.03±0.25 |
| PRBC before scanner | 712 | 0.64±1.74 |
| FFP before scanner | 712 | 0.40±1.24 |
| Prehospital freeze dried plasma | 712 | 10(1%) |
| Transfusion during initial care | 712 | 173 (24%) |
| PRBC after 24 hours | 712 | 2.1±5.0 |
| FFP after 24 hours | 712 | 1.6±4.1 |
| CP after 24 hours | 712 | 0.3±1.4 |
| Massive transfusion | 712 | 45 (6%) |
| Spine fracture and/or spinal cord injury | 712 | 230 (32%) |
| Spinal cord injury with neurological deficit | 712 | 41 (6%) |
| Highest spinal cord injury | 41 |  |
| Cervical |  | 11 (27%) |
| Thoracic |  | 20 (49%) |
| Lombal |  | 9 (22%) |
| Sacral |  | 1 (2%) |
| Hemothorax | 712 | 182 (25%) |
| Unilateral lung contusion | 712 | 56 (8%) |
| Bilateral lung contusion | 712 | 129 (18%) |
| Unilateral or bilateral lung contusions | 712 | 185 (26%) |
| Rib flail Chest | 712 | 75 (9%) |
| Surgery or interventional radiology during the first 24hours | 712 | 457 (64%) |
| Gas tamponade decompression | 205 | 11 (5%) |
| Chest tube | 712 | 84 (12%) |
| Thoracic surgery | 712 | 24 (3%) |
| Rib fixation | 712 | 8 (1%) |
| Abdominal surgery | 167 | 26 (16%) |
| Prehospital tranexamic acid | 204 | 51 (25%) |
| Prehospital antibiotic administration | 712 | 91 (13%) |
| Thoracic radiography | 712 | 405 (57%) |
| Prehospital intubation | 712 | 667 (93%) |
| Initial PaO2 | 682 |  |
| *60<X<150* |  | 239 (35%) |
| *<60* |  | 43 (6%) |
| *>150* |  | 400 (59%) |
| Minimal PaO2/FiO2 | 166 | 220±133 |
| Day of first pneumonia | 192 | 2.4±0.7 |
| Day of first pneumonia | 192 |  |
| *2* |  | 136 (71%) |
| *3* |  | 36 (20%) |
| *4* |  | 12 (7%) |
| *5* |  | 3 (2%) |
| Number of intubations | 499 | 1.2±0.6 |
| Number of intubations | 499 |  |
| *1* |  | 414 (83%) |
| *2* |  | 70 (14%) |
| *3* |  | 10 (2%) |
| *4* |  | 3 (<1%) |
| *7* |  | 1 (<1%) |
| *8* |  | 1 (<1%) |
| Mechanical ventilation (days) | 687 | 6 [ 2 ; 19 ] |
| Tracheotomy | 698 | 150 (21%) |
| ARDS | 712 | 144 (20%) |
| VV ECMO | 712 | 10 (1%) |
| Infection | 712 | 256 (36%) |
| Urinary sepsis | 712 | 27 (4%) |
| Abdominal sepsis | 712 | 29 (4%) |
| Neuromeningeal sepsis | 712 | 5 (<1%) |
| Operating site sepsis | 712 | 51 (7%) |
| Medical device related sepsis | 712 | 72 (10%) |
| Septic shock |  | 61 (9%) |
| ICU LOS (days) | 712 | 13 [6 ; 29] |
| ICU LOS (days) if death occured in ICU | 71 | 18 [ 8 ; 40 ] |
| Hospital LOS | 663 | 28 [13 ; 48] |
| Death in ICU | 712 | 71 (10%) |
| Day 30 mortality | 692 | 52 (8%) |
| Withdrawal or withholding of life-sustaining therapy decision | 712 | 57 (8%) |
| Organ removal procedure | 712 | 3 (<1%) |

*VAP: Ventilator-associated pneumonia, BMI : Body Mass Index; ASA : American society of anesthesiologists; IGS : Index de gravité Simplifié; SOFA: Sepsis-related Organ Failure Assessment; AIS : Abbreviated injury Score; ISS : Injury Severity Score; GCS : Glasgow Coma Scale; SBP ; Systolic blood pressure; DBP : Diastolic blood pressure; HR : heart Rate; SpO_2_ : peripheral capillary oxygen saturation; PRBC : packed red blood cells; FFP : Fresh Frozen Plasma; CP : concentrate of platelets ; EtCO_2_ : End Tidal carbon dioxyde, pH : potentiel hydrogene; PaO_2_ : arterial oxygene partial pressure; PaCO_2_: arterial carbon dioxyde partial pressure; FiO_2_ : Fraction of inspired oxygen; HCO_3_^-^ : blood bicarbonates level; ARDS : Acute Respiratory distress Syndrom; VV ECMO : Veno-venous Extra Corporeal Membrane Oxygenation; ICU LOS : Intensive care unit Length of Stay ; LOS : length of stay ; ICU : Intensive Care Unit.*
