# Supplementary material for: Risk factors for early suspected ventilator-associated pneumonia in severe thoracic blunt trauma patient: A French national cohort study
Source: PLoS One. 2025 May 27;20(5):e0324120. doi: 10.1371/journal.pone.0324120 (PMC12112400; doi:10.1371/journal.pone.0324120)
Supplement: Attachment — Submitted filename: response to reviewer.docx [file pone.0324120.s004.docx]

6. Review Comments to the Author

Please use the space provided to explain your answers to the questions above. You may also include additional comments for the author, including concerns about dual publication, research ethics, or publication ethics. (Please upload your review as an attachment if it exceeds 20,000 characters)

Reviewer #1: Thank you for your corrections. As you say in the discussion, the declarative nature of the diagnosis, the absence of an adjudication committee and the absence of any microbiological features represent significant limitations.

Reviewer #2: I would like to thank the authors for addressing my comments and revising the manuscript accordingly. I appreciate the effort made to improve the clarity and precision of the text.
However, I noticed that the term "suspected VAP" has been incorrectly replaced with "suggested VAP" throughout the manuscript. The term "suggested VAP" is not appropriate and should be corrected to "suspected VAP" everywhere. Additionally, the title should clearly indicate that the study focuses on early suspected VAP.

***Dear reviewer, this mistake has been corrected everywhere in the text and tables***

Some other points remain unaddressed:
• The conclusion of the abstract still omits ASA score from the list of factors independently associated with VAP.

***Dear reviewer, ASA score has been added***

• The abbreviation AIS is still inconsistently defined in the abstract and main text ("acute injury score" vs. "abbreviated injury scale").

***Dear Reviewer, sorry for this omission. It has been corrected***

• The redundancy in "ISS score" has not been fully corrected, as "ISS score" still appears in some instances.

***Dear reviewer, this redundancy has been corrected.***

• "Male sex" should be replaced with "male gender" throughout the manuscript.

***Dear Reviewer, it has been corrected***

Finally, in the second-to-last sentence before the conclusion on page 15, "this results" should be corrected to "this result."

***Dear Reviewer, it has been corrected***

I have no further comments.
I wish the authors much success in their future interventional project, which may further validate the preliminary findings of this study. It’s great to see such work progressing in this field!

Reviewer #3: Thank you for your response to my comments. The authors have addressed all my concerns and strengthened this paper.
